# The Vaginal Community State Types Microbiome-Immune Network as Key Factor for Bacterial Vaginosis and Aerobic Vaginitis

**DOI:** 10.3389/fmicb.2019.02451

**Published:** 2019-10-30

**Authors:** Francesco De Seta, Giuseppina Campisciano, Nunzia Zanotta, Giuseppe Ricci, Manola Comar

**Affiliations:** ^1^Institute for Maternal and Child Health “IRCCS Burlo Garofolo”, Trieste, Italy; ^2^Department of Medicine, Surgery and Health Sciences, University of Trieste, Trieste, Italy

**Keywords:** vaginal microbiome, immune proteins, *Lactobacillus* species, *Bifidobacteria*, vaginal community state types

## Abstract

Regarding bacterial vaginosis (BV), the relevance of the vaginal microbiota to the women’s health fulfills a key role, but knowledge gaps regarding aerobic vaginitis (AV) exist. This study aims to characterize vaginal microbiome and its relationship with the local immune mediators, providing an opportunity to define the link between vaginal commensal microorganisms and opportunistic pathogens in the relation of a given vaginal community state type (CST). A total of 90 vaginal samples from Caucasian asymptomatic women of reproductive age (18–40 years) attending the yearly examination and not reporting any vaginal complaints were retrospectively evaluated for microbiome assessment and immune factor dosage. The samples were tested by the Ion Torrent PGM and the Luminex Bio-Plex technologies for the analysis of microbiome and immune factors, respectively. In our study, the CST classification together with the local immune response profiling represented a good predictive indicator of the vaginal health, suggesting that the predominance of a specific *Lactobacillus* and its relative abundance are pivotal elements to maintain a physiologic status. A vaginal colonization from *Bifidobacterium* may absolve a protective role similar to that of *Lactobacillus*, corresponding to a newly identified CST, although studies are needed to better clarify its clinical significance. Moreover, within each CST, a different pattern of inflammation is activated and orchestrated both by the dominant *Lactobacillus* spp. and by specific non-*Lactobacillus* bacteria and can give insights into the pathogenic mechanisms. In conclusion, this study contributes to the characterization of vaginal dysbiosis, reshaping this concept by taking into consideration the CST profiling, local immune marker, and immune–microbial network.

## Introduction

Vaginal dysbiosis consists of a prolonged deviation from a low-diversity, *Lactobacilli*-dominated microbiome (Van de Wijgert and Jespers, 2017). Molecular studies have identified different types of vaginal dysbiosis, of which the most common and best studied is called bacterial vaginosis (BV), an anaerobic polymicrobial disease associated with subclinical vaginal inflammation (Torcia, 2019). Conversely, dysbiotic states that are associated with clinically overt inflammation include aerobic vaginitis (AV), vaginal candidiasis, and trichomoniasis (Kenyon et al., 2013; Van de Wijgert and Jespers, 2017). The association between dysbiotic microbiota with increased susceptibility to HIV, human papillomavirus (HPV), and other sexually transmitted infections (STIs) and increased risk of pelvic inflammatory disease, preterm birth, and maternal and neonatal infections (Martin et al., 1999; Guo et al., 2012; Kroon et al., 2018) has been observed.

The microbial composition of vagina differs from that of other human surfaces and mucosal sites, characterized by a lower microbial diversity dominated by *Lactobacillus* species. These bacteria, acidifying the vaginal environment, play an important role in local defense (Borges et al., 2014; Aldunate et al., 2015). Most women have a vaginal microbiome dominated by *Lactobacilli*, which are associated with a balanced immune-tolerant vaginal microenvironment, although not all the species equally contributed. To be precise, *Lactobacillus crispatus* does not induce a vaginal mucosal inflammation, and it is also associated with protection from pathogens (Donnarumma et al., 2014). Conversely, *Lactobacillus iners* is much more easily displaced from the vaginal mucosa and often co-occurs with dysbiosis-associated microbes and inflammatory process (Macklaim et al., 2013). A not-well-identified role has been attributed for *Lactobacillus gasseri* and *Lactobacillus jensenii* (Forney et al., 2006; Borges et al., 2014). It has been shown that particular bacterial species are able to colonize both the gastrointestinal and reproductive tract of women, suggesting the rectum as the origin of bacteria commonly colonizing the vagina (Freitas and Hill, 2017). AV is a different vaginal condition with respect to BV, with a specific clinical management and distinct clinical risks (Donders et al., 2011). AV is characterized by a decrease in the amount of *Lactobacillus* but, unlike BV, is accompanied by severe inflammation and the presence of mainly aerobic enteric species, including Group B *Streptococcus* (*Streptococcus agalactiae*), *Enterococcus faecalis*, *Escherichia coli*, and *Streptococcus aureus* (Fredricks et al., 2005; Ghartey et al., 2014; Kaambo et al., 2018). The diagnosis of AV is performed by microscopy on wet mount, using a scoring system. The AV score may indicate normal, intermediate, or severe AV, dominated by an increase grade of inflammation severity (Anahtar et al., 2015). Nevertheless, these methods showed some limitations such as the impossibility of discerning between *Lactobacillus* spp. and bacterial species. Thus, it is difficult to make a differentiated diagnosis between AV and BV, causing the implementation of incorrect treatments, with further consequences including desquamative inflammatory vaginitis, preterm delivery, chorioamnionitis, and funisitis of the fetus during pregnancy (Donders et al., 2002, 2017; Reichman and Sobel, 2014). Considering the relevance of the vaginal microbiota to women’s health and the existing knowledge gap regarding AV, the study of vaginal microbiome and its relationship with the local immune mediators will provide an opportunity to define the link between vaginal commensal microorganisms and opportunistic pathogens in relation to a given vaginal community state type (CST; Kaambo et al., 2018). Such studies will contribute to the characterization of AV and BV dysbiosis and may well inform about the importance of the immune–microbial specific network in identifying dysbiosis.

## Materials and Methods

### Demographics of the Studied Cohort

Ninety vaginal swabs from Caucasian asymptomatic women of reproductive age (18–40 years) were retrospectively evaluated for microbiome assessment and immune factor dosage. Vaginal swabs were performed during the yearly examination from asymptomatic women who did not report any vaginal complaints. The exclusion criteria were >40 years old, menstrual flow, sexual intercourses in the last 3 days, pregnancy, menopause, antibiotic/probiotic therapy in the last 3 months, hormonal therapy, any contraceptive methods (such as condom, pills, vaginal ring, and intrauterine device), and known history of STIs.

### Ethics Statement

The study was approved by the Institutional Scientific Board of the Institute for Maternal and Child Health—IRCCS “Burlo Garofolo” of Trieste, Italy (RC 26/13). All procedures performed in this study involving human participants were in accordance with the ethical standards of the institutional and/or national research committee and with the 1964 Declaration of Helsinki and its later amendments or comparable ethical standards. Informed consent was obtained from all the participants included in the study.

### DNA Extraction and Next-Generation Sequencing (NGS) Library Preparation

Bacterial DNA was extracted using the NucliSENS^®^ easyMAG^®^ system (BioMèrieux, Gorman, NC, United States), following the manufacturer’s instructions, starting from 500 μl and with a final elution volume of 50 μl. Briefly, a 500-base-pair region of the V1–V3 portion of the 16S rRNA gene and, subsequently, the 200-base-pair region of the V3 portion was amplified, as elsewhere described (Campisciano et al., 2018). The V3 amplicon was used for template preparation by the Ion PGM Hi-Q View kit on the Ion OneTouch^TM^ 2 System (Life Technologies, Grand Island, NY, United States) and sequenced using the Ion PGM Hi-Q View sequencing kit (Life Technologies, Grand Island, NY, United States) with the Ion PGM^TM^ System technology. Negative controls, including a no-template control, were processed with the clinical samples.

### Dosage of the Immune Soluble Factors

A soluble concentration of 48 cytokines, chemokines, and growth factors was assessed in duplicate in all 90 vaginal swabs using magnetic bead-based multiplex immunoassays (Bioplex Pro^TM^ human cytokine 21-plex and 27-plex panel, Bio-Rad Laboratories, Milan, Italy) according to the pre-optimized protocol (Zanotta et al., 2019). In brief, the undiluted samples (50 μl) were mixed with biomagnetic beads in 96-well flat-bottom plates, and after incubation for 30 min at room temperature followed by washing plate with Bio-Plex wash buffer, 25 μl of the antibody–biotin reporter was added. After the addition of 50 μl of streptavidin–phycoerythrin (PE) and following incubation for 10 min, the concentrations of the cytokines were determined using the Bio-Plex-200 system (Bio-Rad Corp., United States) and Bio-Plex Manager software (v.6, Bio-Rad). The data were expressed as median fluorescence intensity (MFI) and concentration (pg/ml).

### Bioinformatics Analysis

Raw sequences were analyzed by using QIIME 1.9.1 software (Caporaso et al., 2010). To optimize the operational taxonomic unit (OTU) detection, reads with an average quality score lower than 20, shorter than 150 bp, and with length of homopolymer >6 and primer mismatches >0 were excluded from the analysis. To reduce the risk of including OTUs that were PCR artifacts, all OTUs that occurred in only one sample were removed. The OTUs defined by a 97% of similarity were assigned using the Vaginal 16S rRNA gene Reference Database, which was constructed by Fettweis et al. (2012), using open-reference OTU picking with a uclust clustering tool. To control for differences in sequencing depth between samples, we normalized the read counts by rarefying the otu table biom to a depth of 5,000 reads/sample.

### Statistical Analysis

Stata (v. 13.1) and GraphPad Prism (v. 5) were used for statistical data analysis of the immune soluble factors. The Kruskal–Wallis one-way analysis of variance was used for comparisons between groups. When a significant *p*-value was observed (*p* < 0.05), a multiple comparison test was used to determine which groups were different. To survey the association between microbial identities and the increase or decrease of specific immune factors, the observation_metadata_correlation.py script (with bootstrapped *p*-value assignment and based on the Pearson score) of QIIME was used.

### Accession Numbers

The dataset has been deposited in the National Center for Biotechnology Information (NCBI) Sequence Read Archive (SRA) under the project number SRP152778.

## Results

### Characterization of the Microbial Profile

From the sequencing of the V3 region of the 16S rRNA gene, we obtained a total of 5,683,700 reads (range 1,278–190,380) and a total number of observed OTUs of 11,168 (the reads were clustered into 100 ± 46 OTUs per sample). For the analyses, we rarefied the otu_table.biom to a depth of 5,000 reads/samples, excluding four samples. The two negative controls did not produce an output after the quality filtering. For samples clustering, we used pairwise Bray–Curtis dissimilarities as the input, using Ward’s method for hierarchical clustering. According to the CST classification (Ravel et al., 2011), we identified 18 samples belonging to CST I characterized by *Lactobacillus crispatus*, 13 samples belonging to CST II characterized by *Lactobacillus gasseri*, 28 samples belonging to CST III characterized by *Lactobacillus iners*, and 22 belonging to CST IV depleted of or with low amount of *Lactobacilli*. Three samples showed two dominant *Lactobacilli* at an equal amount, defined as mixed CST. In one sample, *L. crispatus* and *L. gasseri* were present, whereas in the other two samples, *L. crispatus* and *L. iners*. Finally, two remaining samples showed the massive presence of *Bifidobacteria* with respect to the other samples (Table 1). The non-*Lactobacillus* bacteria that were identified within each CST are detailed in Table 2. In particular, in six out of 18 CST I samples, the presence of *Gardnerella vaginalis*, *Ureaplasma parvum*, *Prevotella timonensis*, *Atopobium vaginae*, and *Bifidobacterium bifidum* was spotted. In the remaining samples belonging to CST I, the relative abundance of *L. crispatus* was higher than 90%. In 12 out of 13 CST II and in 10 out of 28 CST III samples, several non-*Lactobacillus* species were present (Table 2). In the remaining samples of CST III, the *L. iners* was above the 98% relative abundance. The 22 CST IV samples showed a highly heterogeneous microbial composition; and the *Lactobacillus* species identified were *L. iners*, *Lactobacillus acidophilus*, *L. gasseri*, *Lactobacillus delbrueckii*, and *Lactobacillus casei.*

**TABLE 1:** The results of the sample grouping.

**Group**	**No. of samples (%)**
CST I	18 (21)
CST II	13 (15)
CST III	28 (33)
CST IV	22 (25)
Mixed CST	3 (3.5)
Bifidobacteria	2 (2.5)

*For the grouping, the output of the taxa_summary.py on the rarefied otu_table.biom (5,000 reads/sample) was used, excluding four samples from the total number (*n* tot = 90). The community state types (CSTs) were named on the basis of the dominant *Lactobacillus*. Mixed CSTs are characterized by two predominant Lactobacilli whereas the Bifidobacteria group by *Bifidobacterium* spp.*

**TABLE 2:** Description of the microbial composition.

**CST**	**Microbiome**
I	13% *Ureaplasma parvum*, **87% *L. crispatus***
I	25% *Gardnerella vaginalis*, **39% *L. crispatus***
I	25% *G. vaginalis*, 1% *U. parvum*, **15% *L. crispatus***
I	1% *Prevotella timonensis*, **22% *L. crispatus***
I	28% *Atopobium vaginae*, 27% *Bifidobacterium breve*, 4% *U. parvum*, **15% *L. crispatus***
I	1% *U. parvum*, **58% *L. crispatus***
II	22% *G. vaginalis*, **75% *L. gasseri***
II	19% *G. vaginalis*, 17% *U. parvum*, **64% *L. gasseri***
II	22% *G. vaginalis*, 12% *U. parvum*, **65% *L. gasseri***
II	9% *G. vaginalis*, 5% *U. parvum*, **83% *L. gasseri***
II	6% *G. vaginalis*, 5% *U. parvum*, **24% *L. gasseri***
II	4% *A. vaginae*, 21% *G. vaginalis*, **65% *L. gasseri***
II	36% *Alloscardovia omnicolens*, 2% *Bifidobacterium bifidum*, **62% *L. gasseri***
II	61% *Escherichia fergusonii*, **39% *L. gasseri***
II	46% *B. breve*, 3% *Streptococcus agalactiae*, **48% *L. gasseri***
II	1% *Gemella haemolysans*, 5% *Staphylococcus haemolyticus*, 9% *Streptococcus australis*, 30% *Streptococcus salivarius*, 1% *Streptococcus sinensis*, 1% *Klebsiella variicola*, **45% *L. gasseri***
II	1% *S. agalactiae*, 4% *Lachnospira pectinoschiza*, 1% *Dialister micraerophilus*, **80% *L. gasseri***
II	30% *B. breve*, 16% *Bifidobacterium scardovii*, 1% *L. pectinoschiza*, **50% *L. gasseri***
II	**14% *Lactobacillus acidophilus*, 1% *L. crispatus*, 34% *Lactobacillus delbrueckii*, 48% *L. gasseri***
III	4% *G. vaginalis*, 1% *U. parvum*, **93% *L. iners***
III	3% *G. vaginalis*, **91% *L. iners***
III	7% *G. vaginalis*, **86% *L. iners***
III	22% *G. vaginalis*, 2% *U. parvum*, 5% *Aerococcus christensenii*, **76% *L. iners***
III	2% *U. parvum*, **96% *L. iners***
III	18% *G. vaginalis*, **81% *L. iners***
III	7% *U. parvum*, 22% *S. agalactiae*, **71% *L. iners***
III	5% *E. fergusonii*, **92% *L. iners***
III	3% *Prevotella melaninogenica*, 4% *Prevotella veroralis*, 1% *S. haemolyticus*, **89% *L. iners***
III	1% *U. parvum*, **97% *L. iners***
III	8% *Prevotella disiens*, 2% *P. timonensis*, 5% *A. christensenii*, 6% *Acidaminococcus fermentans*, 2% *D. micraerophilus*, **4% *L. acidophilus*, 73% *L. iners***
IV	1% *Eggerthella sinensis*, 2% *Prevotella amnii*, 2% *Prevotella shahii*, 5% *P. timonensis*, 85% BVAB2, 2% *Veillonella montpellierensis*
IV	6% *A. vaginae*, 1% *E. sinensis*, 10% *G. vaginalis*, 5% *Sneathia sanguinegens*, 47% *P. timonensis*, 2% *A. christensenii*, 4% *D. micraerophilus*, 6% *V. montpellierensis*, 5% *Parvimonas micra*, **4% *L. iners***
IV	3% *P. bennonis*, 3% *P. somerae*, 6% *P. timonensis*, 1% *Tissierellia coagulans*, 2% *D. propionicifaciens*, 1% *A. tetradius*, 2% *P. ivorii*, 3% *C. ureolyticus*, 74% *E. fergusonii*
IV	10% *A. vaginae*, 3% *E. sinensis*, 10% *G. vaginalis*, 2% *Fusobacterium equinum*, 17% *S. sanguinegens*, 2% *U. parvum*, 11% *Prevotella bivia*, 18% *P. disiens*, 4% *P. timonensis*, 4% *Gemella palaticanis*, 3% *Peptoniphilus stomatis*, 3% *D. micraerophilus*, 8% *V. montpellierensis*, **3% *L. iners***
IV	7% *A. vaginae*, 1% *E. sinensis*, 18% *G. vaginalis*, 18% *S. sanguinegens*, 8% *P. amnii*, 23% *P. timonensis*, 3% *D. micraerophilus*, 4% *V. montpellierensis*, 2% *P. micra*, **9% *L. iners***
IV	3% *A. vaginae*, 94% *G. vaginalis*
IV	14% *A. vaginae*, 2% *E. sinensis*, 34% *S. sanguinegens*, 28% *P. amnii*, 1% *P. timonensis*, 1% *D. micraerophilus*, 6% *V. montpellierensis*, **12% *L. iners***
IV	23% *A. vaginae*, 6% *G. vaginalis*, 44% *P. bivia*, 10% *V. montpellierensis*, **17% *L. iners***
IV	74% *G. vaginalis*, 3% *D. micraerophilus*, **2% *Lactobacillus casei*, 4% *L. gasseri*, 15% *Lactobacillus johnsonii***
IV	12% *A. vaginae*, 9% *G. vaginalis*, 29% *P. bivia*, 3% *Prevotella oris*, 4% *P. timonensis*, 3% *P. stomatis*, 3% *D. micraerophilus*, 2% *Finegoldia magna*, **3% *L. gasseri*, 28% *L. iners***
IV	98% *S. agalactiae*, 1% *D. micraerophilus*
IV	86% *Staphylococcus pasteuri*, 5% *Staphylococcus simiae*, 1% *Enterococcus faecalis*, 3% *S. agalactiae*, 5% *E. fergusonii*
IV	92% *A. vaginae*, 4% *E. fergusonii*
IV	31% *E. fergusonii*, 34% *Klebsiella granulomatis*, 33% *K. variicola*,
IV	2% *A. vaginae*, 60% *Streptococcus anginosus*, 37% *S. simiae*
IV	83% *Corynebacterium pyruviciproducens*, 1% *Fusobacterium naviforme*, 2% *Prevotella pallens*, 3% *D. micraerophilus*, 3% *E. fergusonii*
IV	3% *S. anginosus*, 6% *A. omnicolens*, 1% *Bifidobacterium longum*, **18% *L. acidophilus*, 70% *L. delbrueckii bulgaricus***
IV	83% *Citrobacter braakii*, 2% *E. fergusonii*, 7% *K. granulomatis*, 2% *K. variicola*, 4% *Staphylococcus blattae*, 2% *Pseudomonas aeruginosa*
IV	77% *Staphylococcus massiliensis*, 4% *Anaerococcus hydrogenalis*, 15% *F. magna*, 3% *E. fergusonii*
IV	30% *S. anginosus*, 38% *A. omnicolens*, 27% *P. bivia*, 6% *D. micraerophilus*
IV	35% *A. omnicolens*, 38% *S. haemolyticus*, 2% *Staphylococcus lugdunensis*, 2% *S. simiae*, 7% *S. agalactiae*, 8% *D. micraerophilus*, 4% *Aureimonas altamirensis*
IV	8% *P. disiens*, 2% *P. timonensis*, 5% *A. christensenii*, 6% *A. fermentans*, 2% *D. micraerophilus*, **4% *L. acidophilus*, 73% *L. iners***
I/II	21% *A. vaginae*, 11% *P. bivia*, 1% *L. pectinoschiza*, 23% *E. fergusonii*, **24% *L. gasseri*, 15% *L. crispatus***
I/III	**46% *L. crispatus*, 52% *L. iners***
I/III	**31% *L. crispatus*, 68% *L. iners***
Bifido	98% *B. breve*
Bifido	19% *B. breve*, 2% *G. vaginalis*, 1% *L. pectinoschiza*, **60% *L. delbrueckii***

*The relative abundances of the bacterial species are the output of the taxa_summary.py script on the rarefied out_table.biom (5,000 reads/sample). For brevity, the samples belonging to CST I in which *Lactobacillus crispatus* and samples belonging to CST III in which *Lactobacillus iners* were uniquely identified are omitted. Community state type I (CST I) is characterized by *L. crispatus*, CST II by *Lactobacillus gasseri*, and CST III by *L. iners*, and CST IV is not characterized by a *Lactobacillus*. Bifido, bifidobacteria. In bold, the identified Lactobacilli species.*

### Significant Associations Between Immune Soluble Factors and Bacteria

**FIGURE 1:**
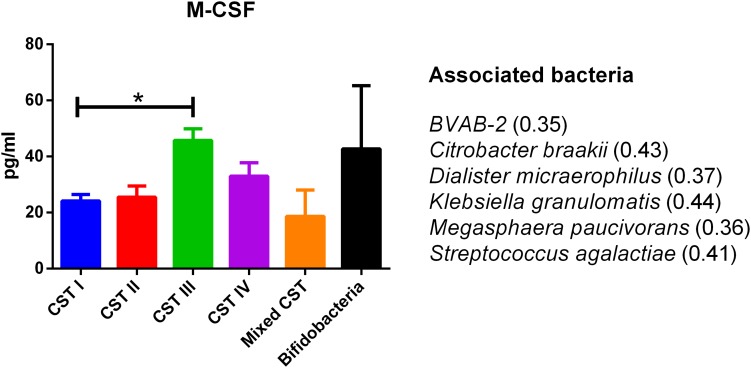
Generic marker of dysbiosis. According to the Pearson correlation score, M-CSF significantly (FDR *p*-value <0.001) correlated with several microorganisms. The association was calculated by means of the observation_metadata_correlation.py script on the rarefied otu_table.biom (5,000 reads/sample). The amount of M-CSF also varied based on the community state type (CST). The comparisons were performed by means of a Kruskal–Wallis one-way analysis of variance. When a significant *p*-value was observed (*p* < 0.05), a multiple comparison test was used to determine which groups were different. The data are shown as the mean value ±standard error of the mean (SEM). Pearson scores for every bacterial species are shown in brackets. FDR, false discovery rate; M-CSF, macrophage colony-stimulating factor. ^∗^*p* < 0.05.

**FIGURE 2:**
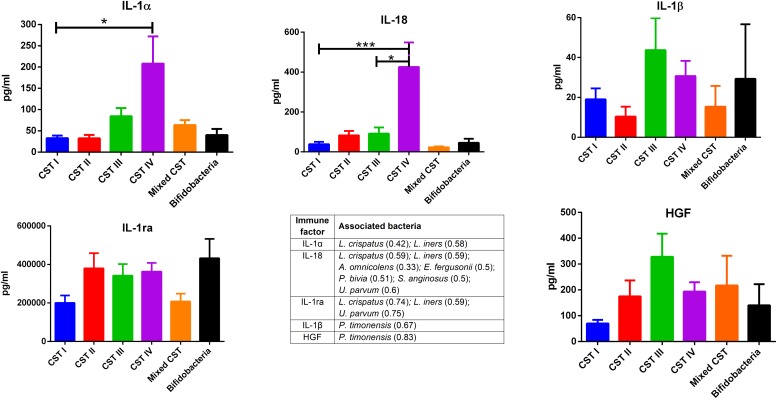
Inflammasome-dependent pathway. According to the Pearson correlation score, components belonging to the inflammasome complex significantly (FDR *p*-value <0.001) correlated with several microorganisms. The association was calculated by means of the observation_metadata_correlation.py script on the rarefied otu_table.biom (5,000 reads/sample). The amount of IL-1α and IL-18 varied based on the community state type (CST). The comparisons were performed by means of a Kruskal–Wallis one-way analysis of variance. When a significant *p*-value was observed (*p* < 0.05), a multiple comparison test was used to determine which groups were different. The data are shown as the mean value ± standard error of the mean (SEM). Pearson scores for every bacterial species are shown in brackets. FDR, false discovery rate. ^∗^*p* < 0.05, ^∗∗∗^*p* < 0.001.

**FIGURE 3:**
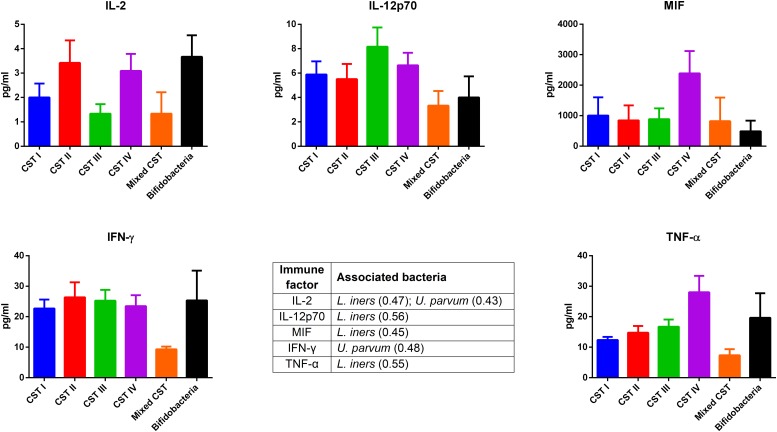
Inflammasome-independent pathway. According to the Pearson correlation score, some pro-inflammatory soluble immune factors (FDR *p*-value <0.001) correlated with *Lactobacillus iners* and *Ureaplasma parvum*. The association was calculated by means of the observation_metadata_correlation.py script on the rarefied otu_table.biom (5,000 reads/sample). The amount of these molecules did not vary based on the community state type (CST). The comparisons were performed by means of a Kruskal–Wallis one-way analysis of variance. When a significant *p*-value was observed (*p* < 0.05), a multiple comparison test was used to determine which groups were different. The data are shown as the mean value ±standard error of the mean (SEM). Pearson scores for every bacterial species are shown in brackets. FDR, false discovery rate.

The concentrations of soluble immune proteins were measured in all 90 vaginal swabs to explore the changes of local immune response associated with different microbial compositions, observed in the different CST cohorts. As shown in Figure 1, macrophage colony-stimulating factor (M-CSF) was correlated to several microorganisms (observation_metadata_correlation.py script, *p* < 0.05), including BVAB2, *Citrobacter braakii*, *Dialister micraerophilus*, *Klebsiella granulomatis*, *Megasphaera paucivorans*, and *Streptococcus agalactiae.* Moreover, the highest amount of M-CSF was observed in CST III, and it was significantly modulated compared with that in CST I (*p* < 0.05). Figure 2 shows the inflammasome-dependent immune network associated with *L. crispatus*, *L. iners*, *Alloscardovia omnicolens*, *Escherichia fergusonii*, *Prevotella bivia*, *Streptococcus anginosus*, *U. parvum*, and *P. timonensis*. CST IV showed a significant increase of two pro-inflammatory cytokines, interleukin (IL)-1α (*p* < 0.05) and IL-18 (*p* < 0.001), compared to the CST I. Only the concentration of IL-18 resulted significantly higher in CST IV compared with CST III (*p* < 0.05). The anti-inflammatory cytokine IL-1ra resulted in an association with *L. crispatus*, *L. iners*, and *U. parvum* (Figure 2). The inflammatory proteins IL-1β and hepatocyte growth factor (HGF) showed an increased trend level in the CST III cohort, and these resulted in an association only with *P. timonensis* (Figure 2). Conversely, Figure 3 shows the inflammasome non-dependent immune network associated with *Lactobacillus iners* and *U. parvum* (Pearson pval_fdr < 0.001). Although no immune protein of this panel showed a statistically significant concentration difference, we observed a higher level of both immune factors macrophage migration inhibitory factor (MIF) and tumor necrosis factor alpha (TNF-α) in CST IV with respect to other cohorts, and both were associated with *L. iners.*

## Discussion

The introduction of the non-culture-based techniques has highlighted a broad spectrum of vaginal microbiome composition in women of reproductive age (Campisciano et al., 2017). In our study, the CST classification (Ravel et al., 2011) together with the profiling of the local immune response represented a good predictive indicator of the vaginal health, suggesting that not only the predominance of a specific *Lactobacillus* but also its relative abundance were pivotal elements to maintain a physiologic status. To be precise, for *Lactobacillus crispatus* and *Lactobacillus iners*, a relative abundance >90% and >98%, respectively, seems to be required to exclude non-commensal microorganisms. Conversely, in our cohort, *Lactobacillus gasseri* is often accompanied with commensal and non-commensal microorganisms, despite its relative abundance. Furthermore, the importance of the *Lactobacillus* species and of its relative abundance is also supported by the absence or the low amount of *Lactobacilli* in the samples belonging to CST IV, also known as the BV-like vaginal microbiome (Torcia, 2019). In two samples of our series, we observed a vaginal microbiota characterized by a massive colonization of *Bifidobacterium* compared with that of the other samples. It is known that *Bifidobacteria* are able to equally colonize the vagina and the gut, where they exert beneficial roles, such as lactic acid production (Sugahara et al., 2015). A recent study has identified vaginal profiles dominated by *Bifidobacterium* in healthy reproductive-aged women, suggesting a potential protective role of these bacteria similar to that of *Lactobacillus* (Freitas and Hill, 2017). Therefore, the presence of *Bifidobacterium* in place of *Lactobacillus* could correspond to a newly identified CST. Although, owing to the paucity of samples with *Bifidobacterium* colonization in our cohort, studies are needed to better clarify its clinical significance.

In our series, the relationship of microbiome composition with immune mediators showed the significant increase in the concentration of the protein M-CSF in CST III with respect to CST I (*p* < 0.05), although this protein was associated with pathogens identified from both CST III and CST IV cohorts (Figure 1). As M-CSF induces the proliferation of monocytes/macrophages and stimulates their phagocytic activity, it seems to represent a non-specific marker of dysbiosis. Nevertheless, the bacterial microbiome composition modulates alternative specific responses, one dependent on the inflammasome complex (Figure 2) and one on the inflammasome-independent pro-inflammatory cytokine secretion (Figure 3), underlining its key role in activating different grades of inflammation. On this way, the commensals *L. crispatus*, *L. iners*, and *Ureaplasma parvum* are associated with the pro-inflammatory inflammasome molecules IL-1α and IL-18 concomitantly with the antagonist IL-1ra, generating a balance between anti-inflammatory and pro-inflammatory response. This equilibrium is unbalanced by the presence of pathogens, which diverts it toward the inflammation. Pathogens such as *Alloscardovia omnicolens*, *Escherichia fergusonii*, *Prevotella bivia*, and *Streptococcus anginosus*, usually considered bacteria responsible of AV, are associated with IL-18 but not with the anti-inflammatory molecule (Strömbeck et al., 2007; Elovitz et al., 2019; Han et al., 2019). Although this mechanism is common to several pathogens, a specific pattern is described for *Prevotella timonensis*, which induces the release of IL-1β, another inflammasome molecule that is known to increase the amount of HGF (Lönn et al., 2014; Bao et al., 2015). Several bacteria, such as *Pseudomonas aeruginosa* and *Helicobacter pylori*, hijack the HGF cascade signaling to establish a comfortable environment for the infection (Strömbeck et al., 2007). Thus, in the vaginal environment, the dosage of HGF can be considered a biomarker of the presence of *Prevotella* (Figure 2). In this study, a massive colonization of *L. iners* and *U. parvum* has been observed that, although vaginal commensals, can induce a baseline inflammation through an inflammasome-independent pathway (Figure 3). To be precise, *U. parvum* is associated with IL-2, which induces the release of interferon gamma (IFN-γ), and *L. iners* is additionally associated with IL-12p70, which stimulates the increased amount of TNF-α (Kasprzykowska et al., 2014). *L. iners* is also associated with MIF, which is known to activate T-cell proliferation and to stimulate the release of TNF-α and IL-2 (Liang et al., 2018). This mechanism could explain why many authors report that a vaginal microbiota belonging to CST III, dominated by *L. iners*, has a higher baseline inflammation with respect to that of CST I and CST II (Ravel et al., 2011; Kacerovsky et al., 2015). Moreover, the increasing trend of pro-inflammatory factors such as MIF and TNF-α in CST IV (Figure 3), although not significant, might indicate the establishment of an inflammatory state associated with the absence or the low amount of *Lactobacilli*, as it has been reported for BV. Therefore, as already disclosed by several studies, the total *Lactobacilli* load in the vagina without considering the bacterial species is not an accurate parameter to assert the health status (Biagi et al., 2009; Jespers et al., 2012). Based on this observation, the introduction of CSTs has led to the comprehension that the species of *Lactobacilli* differently and not equally exert a protective role in the invasion of pathogens (Parolin et al., 2018). In addition, we demonstrate that within each CST, a different pattern of inflammation is activated and orchestrated both by the dominant *Lactobacillus* spp. and by specific non-*Lactobacillus* bacteria and can give insights into the pathogenic mechanisms. In conclusion, we should reshape our concept of dysbiosis by taking into consideration the CST profiling, local immune marker, and immune–microbial network.

## Data Availability Statement

The datasets generated for this study are available on request to the corresponding author.

## Ethics Statement

The studies involving human participants were reviewed and approved by IRCCS BURLO GAROFOLO. The patients/participants provided their written informed consent to participate in this study.

## Author Contributions

MC, FD, and GR contributed to the conception and design of the study, revised the manuscript critically for important intellectual content, and provided approval for publication of the content. GC and NZ organized the database and performed the statistical analysis. GC wrote the first draft of the manuscript. NZ wrote sections of the manuscript. All authors contributed to manuscript revision and read and approved the submitted version.

## Conflict of Interest

The authors declare that the research was conducted in the absence of any commercial or financial relationships that could be construed as a potential conflict of interest.
